# Tropism of AAV Vectors in Photoreceptor-Like Cells of Human iPSC-Derived Retinal Organoids

**DOI:** 10.1167/tvst.11.4.3

**Published:** 2022-04-04

**Authors:** Michelle E. McClements, Hannah Steward, William Atkin, Emily Archer Goode, Carolina Gándara, Valeria Chichagova, Robert E. MacLaren

**Affiliations:** 1 Nuffield Laboratory of Ophthalmology, Department of Clinical Neurosciences, University of Oxford, and Oxford University Hospitals NHS Foundation Trust, NIHR Biomedical Research Centre, Oxford, UK; 2 Newcells Biotech Limited, Newcastle upon Tyne, UK

**Keywords:** AAV, retinal organoids, photoreceptor, gene therapy, inherited retinal disease

## Abstract

**Purpose:**

To expand the use of human retinal organoids from induced pluripotent stem cells (iPSCs) as an in vitro model of the retina for assessing gene therapy treatments, it is essential to establish efficient transduction. To date, targeted transduction of the photoreceptor-like cells of retinal organoids with adeno-associated virus (AAV) vectors has had varied degrees of success, which we have looked to improve in this study.

**Methods:**

Retinal organoids were differentiated from iPSCs of healthy donors and transduced with reporter AAV containing a CAG.GFP, CAG.RFP, GRK1.GFP, or EFS.GFP transgene. Capsid variants assessed were AAV5, AAV2 7m8, AAV2 quad mutant, AAV2 Y444F, and AAV8 Y733F. At 27 days post-transduction, retinal organoids were assessed for reporter expression and viability.

**Results:**

The short intron-less elongation factor 1 alpha (EFS) promoter provided minimal reporter expression, whereas vectors containing the CAG promoter enabled transduction in 1% to 37% of cells depending on the AAV serotype; the AAV2 quad mutant (average 19.4%) and AAV2 7m8 (16.4%) outperformed AAV5 (12%) and AAV8 Y733F (2.1%). Reporter expression from rhodopsin kinase (GRK1) promoter transgenes occurred in ∼5% of cells regardless of the serotype. Positive co-localization with recoverin-expressing cells was achieved from all GRK1 vectors and the CAG AAV2 quad mutant variant. Treatment with the AAV vectors did not influence retinal organoid viability.

**Conclusions:**

Reliable transduction of the photoreceptor-like cells of retinal organoids can be readily achieved. When using a CAG-driven transgene, transduction of a broad range of cell types is observed, and GRK1 transgenes provide a more restricted expression profile locating to the outer layer of photoreceptor-like cells of retinal organoids.

**Translational Relevance:**

This study expands the AAV capsid and transgene options for preclinical testing of gene therapy in iPSC-derived human retinal organoids.

## Introduction

Gene therapy treatments for inherited retinal diseases have developed rapidly in the past decade, with dozens of clinical trials for different disorders currently ongoing (https://clinicaltrials.gov). The vast majority of such trials use adeno-associated viral (AAV) vectors to deliver a transgene of interest, and they range in scope from gene supplementation for single gene disorders, such as X-linked retinitis pigmentosa^1^ and choroideremia,^2^ to the more complex age-related macular degeneration.^3^ All vectors undergo preclinical testing that typically involves a combination of in vitro and in vivo studies to confirm not only that the vectors generate the desired expression products but also that they offer a degree of efficacy and an absence of toxicity. However, the first assessment of the vectors in human-relevant eye tissue commonly occurs during phase I clinical trials. Although human retinal tissue can be extracted and maintained in culture from postmortem^4^^,^^5^ and retinectomy^6^ samples, AAV testing in such samples can be of limited use. Confirmation of vector activity in human retinal explants is of value, but the window of use is often restricted, and the health of the tissue can be questionable; therefore, the observed success of vector transduction can only be taken as supplementary to other data.^7^ The now-established development of human retinal organoids from embryonic stem cells^8^ but more commonly from induced-pluripotent stem cells (iPSCs)^9^^–^^12^ is likely to have a significant impact on preclinical testing of future gene therapy vectors. This is due to the encouraging laminar structure of the retinal organoids, which strongly mimics both the retinal structure and the transcriptome profile of the mammalian eye,^13^ thus allowing researchers to transduce human cells that imitate the retina.^5^^,^^14^^–^^16^ Not only can vector expression profiles be determined but also subsequent testing of treatment effects such as changes to cell morphology and function.^17^^,^^18^

A critical impact of retinal organoid development is the ability to generate them from patient samples. Developing such models from patients with known genetic mutations and comparing these to retinal organoids from wild-type samples have given insights into retinal disease.^18^^–^^25^ Furthermore, these retinal organoids can be treated with gene therapy vectors to correct the disease-related features.^18^^,^^21^^,^^24^ Such assessment of gene therapy vectors prior to clinical trial could prove invaluable, particularly as the nature of such treatments evolves to focus on methods based on clustered regularly interspaced short palindromic repeat (CRISPR)–CRISPR-associated (Cas) systems.^26^^–^^29^ Unlike current gene supplementation strategies, CRISPR-Cas systems will be mutation specific. As CRISPR-Cas gene therapies develop for the treatment of inherited retinal disease, the use of in vivo testing may become limited to assessments of toxicity and off-target effects, with treatment of retinal organoids from patient-generated iPSCs providing direct evidence of clinical vector efficacy.

With the impact of human retinal organoids on gene therapy programs already evident and only likely to increase in the coming years, there is a need for vector capsid and transgene combinations that efficiently transduce particular desired cell types. The majority of inherited retinal diseases are caused by mutations in genes expressed in the photoreceptor rod and cone cells, which are therefore the primary targets of current clinical trials. It is generally considered that the outer layer of cells of retinal organoids represent photoreceptor-like cells, and indeed these typically express markers such as neural leucine zipper protein, recoverin, arrestin, rhodopsin, and cone opsin.^9^^–^^12^ AAV transduction of human retinal organoids has been previously reported, with AAV2 7m8 so far showing the best rates of transduction in retinal organoids.^5^^,^^14^^,^^15^ AAV5 has been used to successfully assess *RP2* gene therapy of human retinal organoids, but it was required at a relatively high dose.^18^ Other capsid variants have been tested on retinal organoids but have had minimal transduction success, including AAV2, AAV8, AAV8 Y733F, and AAV9.^14^^,^^15^^,^^24^ Modified variants of AAV9 have also been shown to provide relatively good transduction profiles.^16^ Critically, the transduction profiles of AAV vectors have been predominantly achieved with the strong ubiquitous promoters cytomegalovirus (CMV) or CMV early enhancer/chicken beta actin (CAG), with limited transduction achieved using the photoreceptor-specific rhodopsin kinase (GRK1) promoter or indeed other photoreceptor promoter options of PR2.1 (L- and M-cone opsin) and rhodopsin (RHO).^14^

We assessed a broad range of transgene and capsid variants to identify the combinations most suited for targeting the photoreceptor-like cells of retinal organoids. For this reason, AAV ShH10 (an AAV6 variant) was not selected despite previous retinal organoid transduction success,^14^ as it has been shown to favor Müller glia transduction.^30^ In contrast, AAV2, AAV5, and AAV8 variants are more commonly used to achieve photoreceptor cell targeting and have been used in clinical trials^31^; therefore, such variants were the focus of this study.

## Materials and Methods

### Human iPSC Culture

The iPSC line WT3 (SB-AD4)^23^ was cultured in mTeSR1 medium (Stemcell Technologies, Vancouver, Canada) supplemented with 1% penicillin/streptomycin (Thermo Fisher Scientific, Waltham, MA) on plates coated with Matrigel (Corning Inc., Corning, NY). Cell culture medium was replaced daily, and cells were allowed to grow for 4 to 5 days prior to passaging at a ratio of 1:6 with Versene ethylenediaminetetraacetic acid (EDTA; Lonza, Basel, Switzerland).

### Differentiation of Retinal Organoids from Human iPSCs

The iPSCs were differentiated to retinal organoids based on a previously described method.^32^ Briefly, iPSCs were dissociated to single cells using Accutase (Thermo Fisher Scientific), and 7000 cells were seeded per well of a U-bottom 96-well plate precoated with Lipidure (Amsbio, Abingdon, UK) in 100-mL mTeSR1 medium supplemented with 10-µM Y-27632 (Chemdea, Ridgewood, NJ), designated day −2. On day 0, 200 mL of differentiation medium was added, comprised of 41% Iscove's Modified Dulbecco's Medium, 41% Ham's F-12 Nutrient Mixture, 15% knockout serum replacement, 1% GlutaMAX supplement, and 1% chemically defined lipid concentrate (all Thermo Fisher Scientific), and 225-µM 1-thioglycerol (Sigma-Aldrich, St. Louis, MO). At day 6, 2.25-nM bone morphogenetic protein 4 (R&D Systems, Minneapolis, MN) was added. On day 18, the medium was changed to Dulbecco's Modified Eagle Medium/Ham's F-12 Nutrient Mixture, 1% GlutaMAX, 10% fetal bovine serum, and 1% N-2 supplement (all Thermo Fisher Scientific); 0.5-µM retinoic acid (Sigma-Aldrich); 0.1-mM taurine (Sigma-Aldrich); and 0.25-µg/mL Fungizone (Thermo Fisher Scientific). After day 120, retinoic acid was removed from the culture medium. The medium was supplemented with 1% penicillin/streptomycin at all times. Each individual retinal organoid was maintained in a single well of a U-bottom 96-well plate.

### AAV Production

Reporter transgenes of CAG.GFP.WPRE.pA, CAG.RFP.pA, GRK1.GFP.pA, or EFS.GFP.WPRE.pA were cloned between AAV2 inverted terminal repeats. The general protocol used for AAV production has been previously detailed.^33^ Briefly, a standard polyethylenimine triple transfection method of HEK293T cells in Corning HYPERFlasks provided transgene plasmid, pAdDeltaF6, and one of the following: pRep2Cap2 Y444F, quad mutant, 7m8, or pRep2Cap8 Y733F. For AAV5 production, pDP5 (PlasmidFactory, Bielefeld, Germany) containing combined helper and *rep* and *cap* genes was used. Cells were harvested 72 hours post-transfection, and lysates were purified using iodixanol gradients with subsequent buffer exchange performed using Amicon Ultra-15 100K filter units (Merck, Kenilworth, NJ). AAV preparations were eluted in no Mg^2+^, no Ca^2+^ phosphate buffered saline with 0.001% Pluronic F68 (Thermo Fisher Scientific). DNase-treated samples were used for quantitative polymerase chain reaction (qPCR) titer with iTaq reagent (Bio-Rad Laboratories, Hercules, CA) and primers binding the pA sequence common to all transgenes (pA qPCR FW: CCAGCCATCTGTTGTTTGCC; pA qPCR RV: GAAAGGACAGTGGGAGTGGC).

### AAV Treatment of iPSC-Derived Retinal Organoids

Following 150 days of differentiation, retinal organoids were treated with 1E+10 genome copies (unless otherwise stated) of AAV in a 50-µL volume. The 50:50 media changes (with no further provision of AAV) were performed every 3 to 4 days. Retinal organoids were harvested for cryosectioning or viability assay at 27 days post-transduction. The number of individual retinal organoids treated per AAV variant were as follows: CAG.RFP AAV5, *n* = 8; AAV2 7m8, *n* = 12; AAV2 quad mutant, *n* = 6; CAG.GFP.WPRE AAV8 Y733F, *n* = 5; EFS.GFP.WPRE AAV2 7m8, *n* = 3; AAV5, *n* = 8; GRK1.GFP AAV8 Y733F, *n* = 14; AAV2 7m8, *n* = 12; and AAV2 Y444F, *n* = 8. Live cell imaging was performed at regular intervals using an EVOS Cell Imaging System (Thermo Fisher Scientific).

### Viability Assay

An adenosine triphosphate (ATP) assay was performed using the CellTiter-Glo 3D Cell Viability kit (Promega, Madison, WI). Each retinal organoid was placed in an individual well of a white Costar 96-well plate (Corning) with 100 µL of media. To each sample, 100 µL of reagent was applied, and the samples were incubated for 30 minutes at room temperature on an elliptical shaker under dark conditions. Luminescence readings were taken using a FLUOstar Omega Plate Reader (BMG Labtech, Ortenberg, Germany). ATP levels for each retinal organoid were determined from an ATP dilution series after being normalized to media-only controls. Data are presented as percentage ATP levels relative to the average ATP levels of four untreated retinal organoids. All treatment groups were *n* = 4. The normal distribution and variance of data were confirmed (Brown–Forsythe and Bartlett's tests), and a one-way analysis of variance (ANOVA) with Dunnett's multiple comparisons was performed on the dataset. For correlation of retinal organoid size with ATP levels, retinal organoid sizes were determined by drawing around a standardized image and using the area measurement function within ImageJ (National Institutes of Health, Bethesda, MD). Area measurements were plotted against ATP levels, and a Pearson *r*-test was performed.

### Cryosectioning and Staining of Retinal Organoids

Retinal organoids were harvested for cryosectioning as previously detailed.^11^ After they were embedded in optimal cutting temperature mounting medium, 10-µm sections were prepared using a cryotome. Slides were washed three times with phosphate-buffered saline (PBS) before a blocking solution was applied for 1 hour at room temperature (10% donkey serum, 1% bovine serum albumin [BSA], 0.3% Triton X-100 in PBS). Samples were then incubated overnight at 4°C with primary antibody solution (1% BSA, 0.3% Triton X-100, PBS) (Table). After three washes in PBS, the secondary antibody solution was applied and left for 2 hours at room temperature (1% BSA, 0.3% Triton X-100, PBS). Sections were washed once with PBS and then incubated with Hoechst (1/2000, PBS) for 30 minutes at room temperature. Finally, slides were rinsed once with PBS and air dried for 30 minutes before Diamond Antifade Mountant (Thermo Fisher Scientific) and a cover slip were applied. Samples were stored at 4°C prior to imaging.

**Table.** Antibodies Used for Immunostaining

Name	Target	Product	Dilution
Rabbit anti-recoverin antibody	Recoverin	AB5585 (Sigma-Aldrich)	1/1000
Rabbit anti-opsin antibody	L-/M-cone opsin	AB5405 (Sigma-Aldrich)	1/200
Mouse anti-rhodopsin antibody [ID4]	RHO	ab5417 (Abcam)	1/500
Rabbit recombinant anti-PKC alpha antibody [Y124]	PKCα	ab32376 (Abcam)	1/200
Goat anti-GFAP antibody	GFAP	ab53554 (Abcam)	1/200
Donkey anti-rabbit IgG H&L (Alexa Fluor 647)	For detection of anti–L/M-cone opsins, anti-recoverin and anti-PKCα	ab150075 (Abcam)	1/400
Donkey anti-mouse IgG H&L (Alexa Fluor 647)	For detection of mouse anti-RHO	ab150107 (Abcam)	1/400
Donkey anti-goat IgG H&L (Alexa Fluor 488 or 568)	For detection of goat anti-GFAP	ab150129 & ab175704	1/400

### Quantitative Analysis of Stained Retinal Organoid Sections

Images of retinal organoid sections were acquired using the 40× objective of a Zeiss LSM 710 confocal microscope (Carl Zeiss Microscopy, Jena, Germany). Z-stacks spanning a 10-µm depth were obtained and combined using the Max Intensity Z-Project function in Fiji/ImageJ (version 2.3.0/1.53f). Channels were split, and the Colocalization Threshold function was used to determine the overlap in transgene expression and marker staining. The Costes method of automatic threshold determination was applied, with output values below zero indicating anti-correlated pixel intensities and values above zero indicating correlation of reporter and marker signal (a value of 1 would indicate perfect correlation of reporter and marker). Statistical analyses were performed using Prism 9.3.1 (GraphPad, San Diego, CA) with comparisons of reporter expression levels achieved by one-way ANOVA with Tukey's or Dunnett's multiple comparisons (stated in figure legends).

## Results

### Live Cell Monitoring of AAV Transduced Retinal Organoids

**Figure 1.**
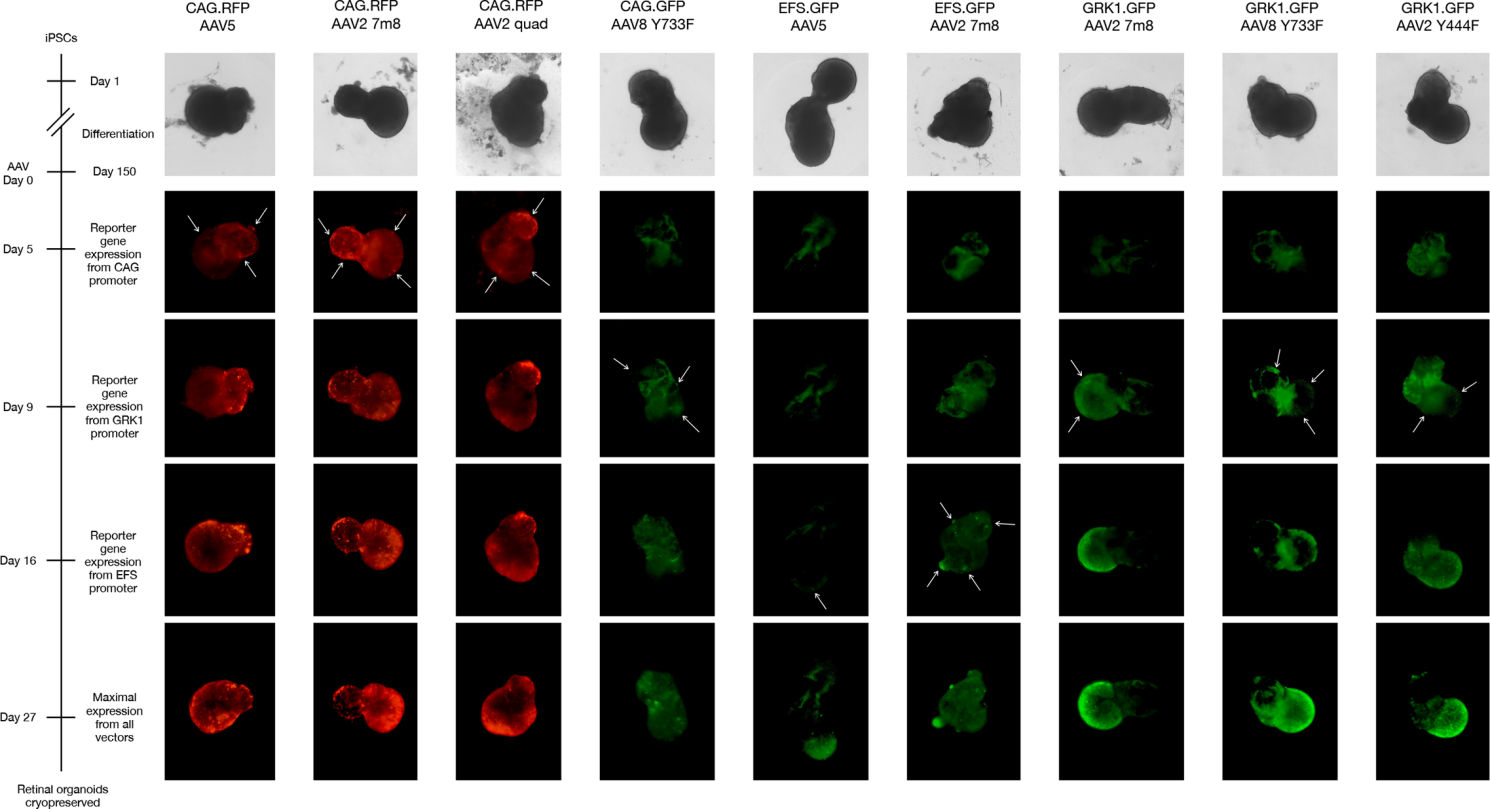
Live cell imaging of reporter gene expression up to 27 days post-transduction. *Arrows* indicate the areas where the onset of reporter gene expression first appeared. Each retinal organoid was treated with 1E+10 genome copies, except for GRK1.GFP AAV8 Y733F, for which 5E+10 genome copies were used. Background fluorescence of live retinal organoids was unavoidable, but reporter expression was evident as higher intensity areas above this background. Further examples of retinal organoids treated per condition are shown in Supplementary Figure S2.

Human retinal organoids derived from the same differentiation batch were treated with AAV at 150 days post-differentiation. Then, 1E+10 genome copies of each AAV were applied to a single retinal organoid unless otherwise stated. As a comparison to other studies, the AAV2 7m8 variant was used with all promoters of interest (CAG, short intron-less elongation factor 1 alpha [EFS], and GRK1), as this capsid has previously been shown to provide consistently good transduction across different studies.^5^^,^^14^^,^^15^ CAG.RFP, CAG.GFP, and EFS.GFP AAV vectors were confirmed to successfully produce desired reporter gene expression in HEK293T cells prior to use on the retinal organoids (Supplementary Fig. S1). Reporter red fluorescent protein (RFP) or green fluorescent protein (GFP) expression was observed in live retinal organoids over 27 days post-transduction (Fig. 1, Supplementary Fig. S2). The first onset of reporter gene expression was observed at 5 days post-transduction from the CAG promoter transgenes packaged in AAV2 7m8, AAV2 quad mutant, and AAV5 capsids. In the AAV8 Y733F vector, CAG-driven reporter expression was not observed until day 9. Early reporter gene expression from the GRK1 promoter transgenes were also noted at day 9 from AAV2 7m8 and AAV2 Y444F vectors and from the AAV8 Y733F vector used at the higher dose of 5E+10 genome copies. Reporter expression from vectors incorporating the EFS promoter was not evident until day 16.

### Marker Analysis of Cell Types Within the Retinal Organoids

Following live cell monitoring, retinal organoids were harvested, fixed, and sectioned. General cell-type populations within the retinal organoids structures were investigated through co-staining of various markers familiar to cell types within the retina: glial fibrillary acidic protein (GFAP) as a marker for Müller glia-type cells; protein kinase C alpha (PKCα), representing bipolar cell populations; recoverin as a generic marker for photoreceptor-like cells; and L-/M-cone opsin or RHO for identification of cone- and rod-like photoreceptor cells, respectively. Recoverin positive cells were the most abundant cell types in the sections analyzed (an average of ∼23% of retinal organoid cells), followed by PKCα (∼8%), L-/M-positive cells (∼7%), and RHO- and GFAP-positive cells (∼6%) (Supplementary Fig. S3). With key cell populations in retinal organoids identified, we proceeded to investigating the general transduction profiles of various AAV vectors.

### Extent of Vector Transduction in Retinal Organoids

**Figure 2.**
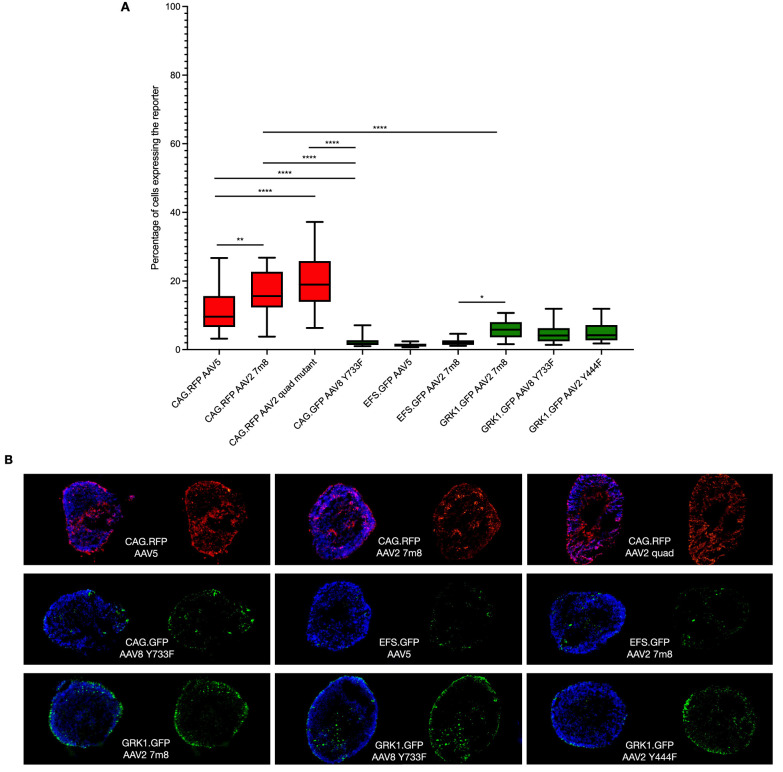
Reporter transgene expression in retinal organoids transduced with different vector types. (**A**) Z-stacks of retinal organoid sections were extracted using 40× magnification and analyzed for reporter-positive cells. Values were obtained from 27 to 35 individual images from at least three different retinal organoids per treatment group. One-way ANOVA with Tukey's multiple comparisons test revealed significant differences among groups. *P = 0.041, **P = 0.003, ****P < 0.0001. Error bars represent minimum/maximum. Example Z-stacks from which the data were extracted are provided in Figures 3 and 4. (**B**) Representative examples of whole retinal organoid sections taken with 10× magnification.

**Figure 3.**
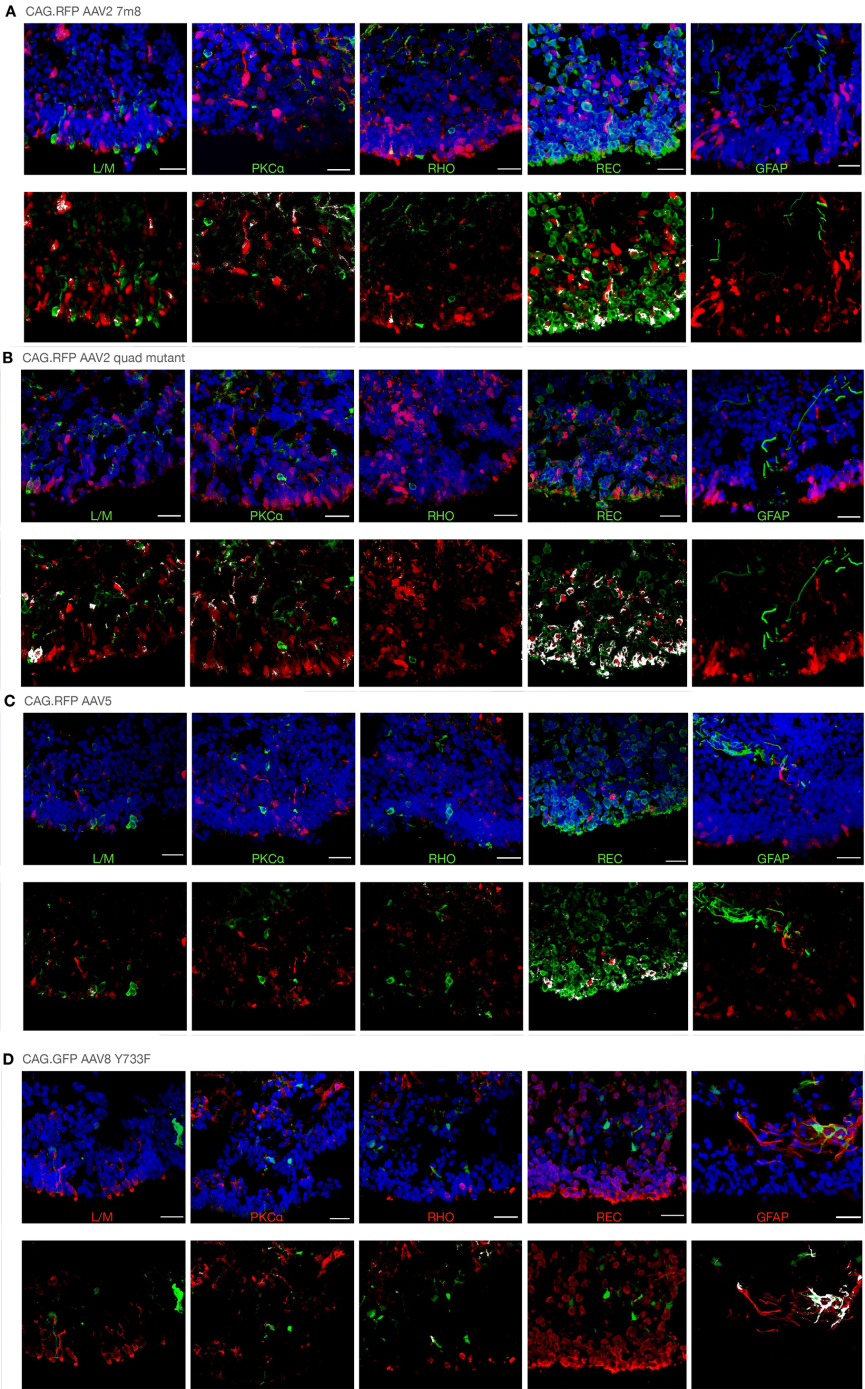
Representative reporter gene expression profiles from CAG transgenes delivered in multiple AAV capsid types. For each sample type, example retinal organoid sections with each marker are shown in the *upper panel*, and only reporter and marker expression is shown in the *lower panel*, for which overlapping signals are highlighted in *white*. (**A**) AAV2 7m8; (**B**) AAV2 quad mutant; (**C**) AAV5; and (**D**) AAV8 Y733F. Each retinal organoid was treated with 1E+10 genome copies. *Scale bars:* 25 µm. Isolated green and red channels are shown in Supplementary Figure S5. GFAP, glial fibrillary acidic protein, a Müller glia cell marker; L/M, long-/medium-wavelength cone opsin, a cone photoreceptor marker; PKCα, protein kinase C alpha, a bipolar cell marker; REC, recoverin, a pan-photoreceptor marker; RHO, rhodopsin, a rod photoreceptor marker.

All transgene and vector combinations tested achieved some degree of reporter gene expression (Fig. 2). As reported in other publications, the CAG promoter enabled transduction across a range of cell types within the retinal organoids (Fig. 3). Using the AAV2 7m8 capsid, transduction was observed in 16.4% ± 6.4% of cells; yet, despite the broad transduction profile, multiple Z-stack section assessments identified no consistent signs of positive co-localizations with any of the markers (Fig. 3A, Supplementary Table S1). For CAG AAV2 quad mutant–treated retinal organoids, transduction was observed in 19.4% ± 7.9% of cells, and a positive correlation was achieved with recoverin-expressing cells (Fig. 3B). When using the AAV5 capsid, 12% ± 7% of cells expressed the reporter transgene in a broad transduction profile that provided no positive correlations with any of the markers.

The transduction levels of retinal organoids treated with CAG AAV2 7m8 and AAV2 quad mutant vectors were not significantly different (*P* = 0.1598), whereas both were significantly different compared to AAV5 (*P* = 0.0030 and *P* < 0.0001, respectively). These three vectors were all significantly better (*P* < 0.0001) at transducing retinal organoids than CAG AAV8 Y733F, for which only 2.1% ± 1.2% of cells expressed the reporter.

The EFS ubiquitous promoter is of interest for gene therapy transgenes thanks to its relatively small size (∼0.2 kb) compared to the CAG (∼1 kb) and CMV (∼0.6 kb) promoters. Although active in immortalized cells lines (Supplementary Fig. S1), minimal reporter gene expression was achieved following transduction in retinal organoids (Supplementary Fig. S4). Reporter positive cells were barely evident when an EFS reporter transgene was packaged in AAV5 (1.4% ± 0.5% of cells), with marginally better transduction achieved using the AAV2 7m8 capsid (2.1% ± 0.9% of cells). These EFS vectors provided significantly reduced levels of reporter expression compared to the equivalent CAG vectors (*P* < 0.0001).

### The GRK1 Promoter Enables Reliable Transgene Expression in Photoreceptor-Like Cells of Retinal Organoids

**Figure 4.**
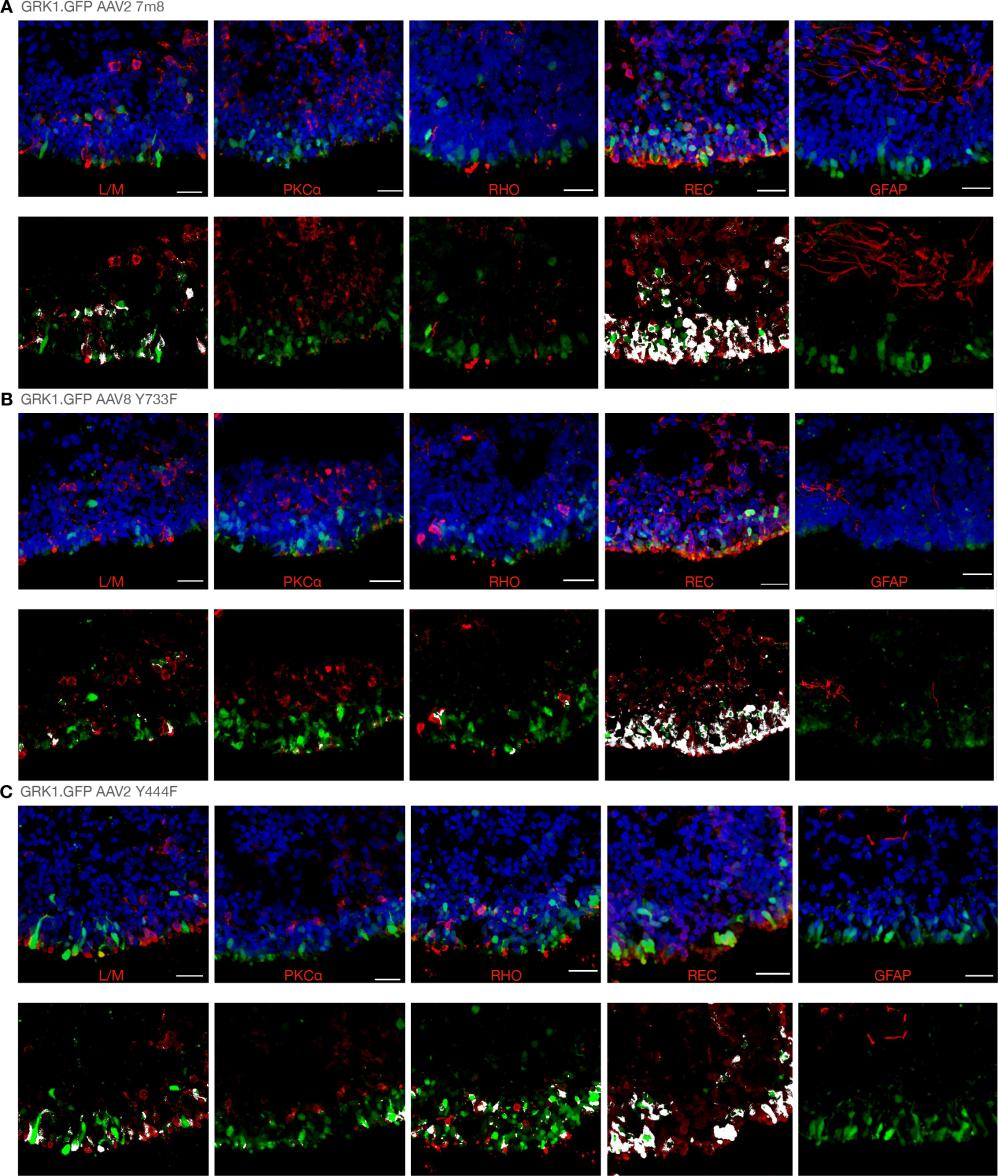
Reporter gene expression profiles from GRK1 transgenes delivered in multiple AAV capsid types. For each sample type, example retinal organoid sections with each marker are shown in the *upper panel*, and only reporter and marker expression is shown in the *lower panel*, for which overlapping signals are highlighted in *white*. (**A**) GRK1.GFP AAV2 7m8; (**B**) GRK1.GFP AAV8 Y733F; and (**C**) GRK1.GFP AAV2 Y444F. Each retinal organoid was treated with 1E+10 genome copies, except in (**B**), where 5E+10 genome copies were used. *Scale bars:* 25 µm. Isolated green and red channels are shown in Supplementary Figure S6.

Previous attempts to achieve AAV transgene expression in retinal organoids using the photoreceptor-specific GRK1 promoter have had limited success.^14^ By contrast, we have achieved consistent levels of reporter gene expression from the GRK1 promoter following transgene delivery with AAV2 7m8 (Fig. 4A), AAV8 Y733F (Fig. 4B), and AAV2 Y444F (Fig. 4C) capsid variants. All vectors provided a clear ring of GFP expression in the outer layer of cells of the transduced retinal organoids (Fig. 2). Reporter gene expression for all GRK1 vectors achieved positive co-localization with recoverin-stained cells (Supplementary Table S1). Reporter expression levels were comparable when delivering the GRK1 transgene with AAV2 7m8 (5.8% ± 2.5% of cells) and AAV2 Y444F (5.3% ± 2.9% of cells). Equivalent transduction levels were also achieved with the AAV8 Y733F capsid (4.7% ± 2.9% of cells), but only when used at the higher dose of 5E+10 genome copies. It was of interest that, when the GRK1.GFP transgene was used at 1E+10 genome copies in the AAV8 Y733F capsid, no reporter expression was achieved (Supplementary Fig. S2), which was in line with the poor expression levels observed from the CAG transgene.

### AAV Treatment of Retinal Organoids Does Not Influence Viability

**Figure 5.**
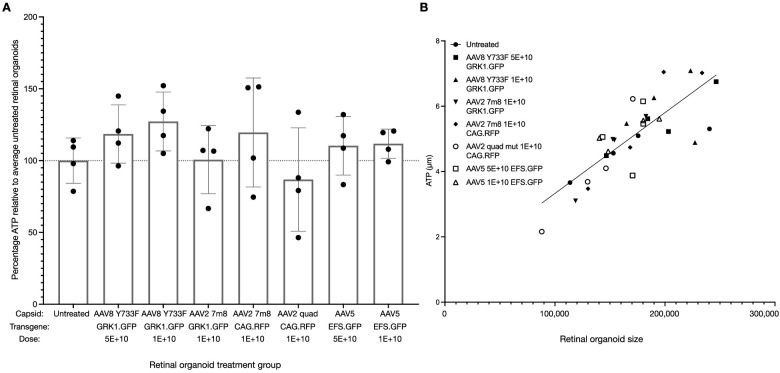
AAV transduction did not influence retinal organoid viability. The average ATP levels from four individual untreated retinal organoids were used to determine the relative percentage ATP activity in each treatment group. (**A**) One-way ANOVA indicated no significant influence of AAV treatment on viability (*F* = 1.107, *P* = 0.3908), and Dunnett's multiple comparisons test revealed no significant differences in any treatment group compared to the untreated group. Error bars represent standard error of the mean. (**B**) ATP levels correlated with retinal organoid size (*r* = 0.8019, *P* < 0.0001, *R*^2^ = 0.6431).

An ATP assay was performed to determine whether AAV treatment influenced retinal organoid viability. Different capsids (AAV5, AAV8 Y733F, AAV2 7m8, and AAV2 quad mutant), transgenes (CAG.RFP, EFS.GFP, and GRK1.GFP), and vector doses (1E+10 or 5E+10 genome copies per retinal organoid) were tested with no significant influence on retinal organoid viability observed (Fig. 5A). ATP levels were not significantly different from untreated samples for any of the treatment groups. ATP levels varied between retinal organoids within each condition, but these differences correlated with the size of retinal organoid (*P* < 0.0001) (Fig. 5B).

## Discussion

The application of retinal organoids in preclinical testing protocols of gene therapy vectors for inherited retinal disease is likely to expand in the future. With treatments shifting from gene supplementation strategies to mutation-dependent CRISPR approaches,^34^ there will be an even greater need to assess efficacy in patient-derived samples. Although correction of patient-derived fibroblast or iPSCs can prove useful for assessments of genome editing, retinal organoids offer the additional prospect of achieving functional and or/morphological outputs.^18^^–^^22^^,^^24^^,^^25^ The most translational preclinical studies would test the exact capsid and transgene combination intended for human use.

In this study, it was apparent that retinal organoids can vary in both size and marker profile, thus influencing the transduction efficiency of samples treated with identical vector preparations. To counter this, we treated multiple retinal organoids per vector type, but with some markers represented in small populations (e.g., GFAP, RHO) achieving positive co-localization with transduced cells would always prove challenging. It was apparent that, although in some retinal organoids transduced cells also expressed certain markers, within the same treatment group transduced cells would not express the marker of interest, causing a negative correlation in co-expression. However, for the pan-photoreceptor marker recoverin, which presented on average in ∼23% of retinal organoid cells, positive co-localization was identified for all vectors containing the GRK1 promoter in addition to the CAG AAV2 quad mutant vector.

Testing of various capsids has been previously presented in retinal organoids, with AAV2, AAV5, AAV8, AAV8 Y733F, and AAV9 proving to have relatively poor expression profiles with CMV- or CAG-driven transgenes.^14^^,^^15^ In contrast, AAV2 7m8 vector transduction has been consistently successful between research groups,^5^^,^^14^^,^^15^ and novel AAV9 variants also show encouraging transduction profiles in retinal organoids.^16^ In this study, we have confirmed the robust transduction capability of AAV2 7m8 in retinal organoids when using the ubiquitous CAG promoter. However, when the ubiquitous EFS promoter was used, minimal reporter gene expression was observed, suggesting that despite the convenient size of this promoter inclusion in gene therapy vectors for inherited retinal disease is not likely to be beneficial.

CAG reporter transgene expression was further achieved when using the AAV5 and AAV2 quad mutant vectors.^35^ As with the AAV2 7m8 capsid, this latter variant has largely been associated with optogenetics studies in the degenerate retina.^36^^,^^37^ In the retinal organoid assessments presented here, reporter transgene expression was achieved in ∼19% of cells, the highest average proportion of any vector tested. Although the AAV2 quad mutant has not yet been used in a clinical trial, the related AAV2 triple mutant variant is currently being tested for safety and efficacy in humans (e.g., NCT02599922), as is the AAV2 7m8 vector (NCT03326336). The AAV2 quad mutant variant may therefore have potential for outer and inner retinal transduction, and it would be of interest to package other transgene variants in this serotype for further comparisons. However, the drawback of the AAV2 quad mutant vector is that its use is proprietary to Applied Genetic Technologies Corporation, which limits its translational application and increases the importance of focusing on less restricted AAV vectors.

Transduction with AAV8 Y733F carrying a CAG transgene provided relatively poor transduction success, in line with previous reports.^14^ This is despite the transgene carrying a woodchuck hepatitis virus posttranscriptional regulatory element (WPRE) not contained in the AAV2 7m8, AAV5, or AAV2 quad mutant CAG transgenes. The AAV8 Y733F CAG vector was tested only at a single dose of 1E+10 genome copies per retinal organoid; yet, it might be that greater transduction efficiency could be achieved at a higher dose (of 5E+10 genome copies), as observed when this serotype was assessed with a GRK1 transgene.

A key aspect of preclinical testing of gene therapy treatments is the ability to test the exact transgene to be incorporated in the clinical vector. The GRK1 promoter has been commonly used in such studies, as it is active in both mouse and human photoreceptor cells and is used in current clinical trials (e.g., NCT03584165, NCT03872479). Future gene therapy vectors for treatments of inherited retinal disease are therefore likely to contain the GRK1 promoter, and being able to test such vectors in retinal organoids would be highly desired. Although previous testing of transgenes containing this promoter has shown limited success,^14^ our study has revealed efficient GRK1-driven reporter gene expression in the photoreceptor-like cells of the retinal organoids. This was achieved with AAV2 7m8, AAV2 Y444F, and AAV8 Y733F capsid variants. The AAV8 Y733F capsid is being used in preclinical studies for photoreceptor targeting due to its advanced transduction rates in vivo over AAV8.^38^^,^^39^ The AAV2 Y444F variant was selected because it is little reported yet, as a single mutant, appears to be more potent than AAV2.^7^^,^^38^^,^^40^ Despite previous reports having minimal success with the GRK1 promoter expression in retinal organoids, we have shown that reliable transduction can be achieved with all three capsid types tested, a result that should be highly relevant for future testing of AAV gene therapy vectors.

In addition to achieving robust levels of expression from AAV vectors in the photoreceptor-like cells of retinal organoids, we have also demonstrated a lack of toxicity from the capsid variants and doses used. We present data that expand the potential of retinal organoids as in vitro models for assessments of AAV gene therapy efficacy for the treatment of inherited retinal diseases.

## Supplementary Material

Supplement 1
