# Supplementary material for: Tropism of AAV Vectors in Photoreceptor-Like Cells of Human iPSC-Derived Retinal Organoids
Source: Transl Vis Sci Technol. 2022 Apr 4;11(4):3. doi: 10.1167/tvst.11.4.3 (PMC8994202; doi:10.1167/tvst.11.4.3)
Supplement: Supplement 1 [file tvst-11-4-3_s001.pdf]

Supplementary material

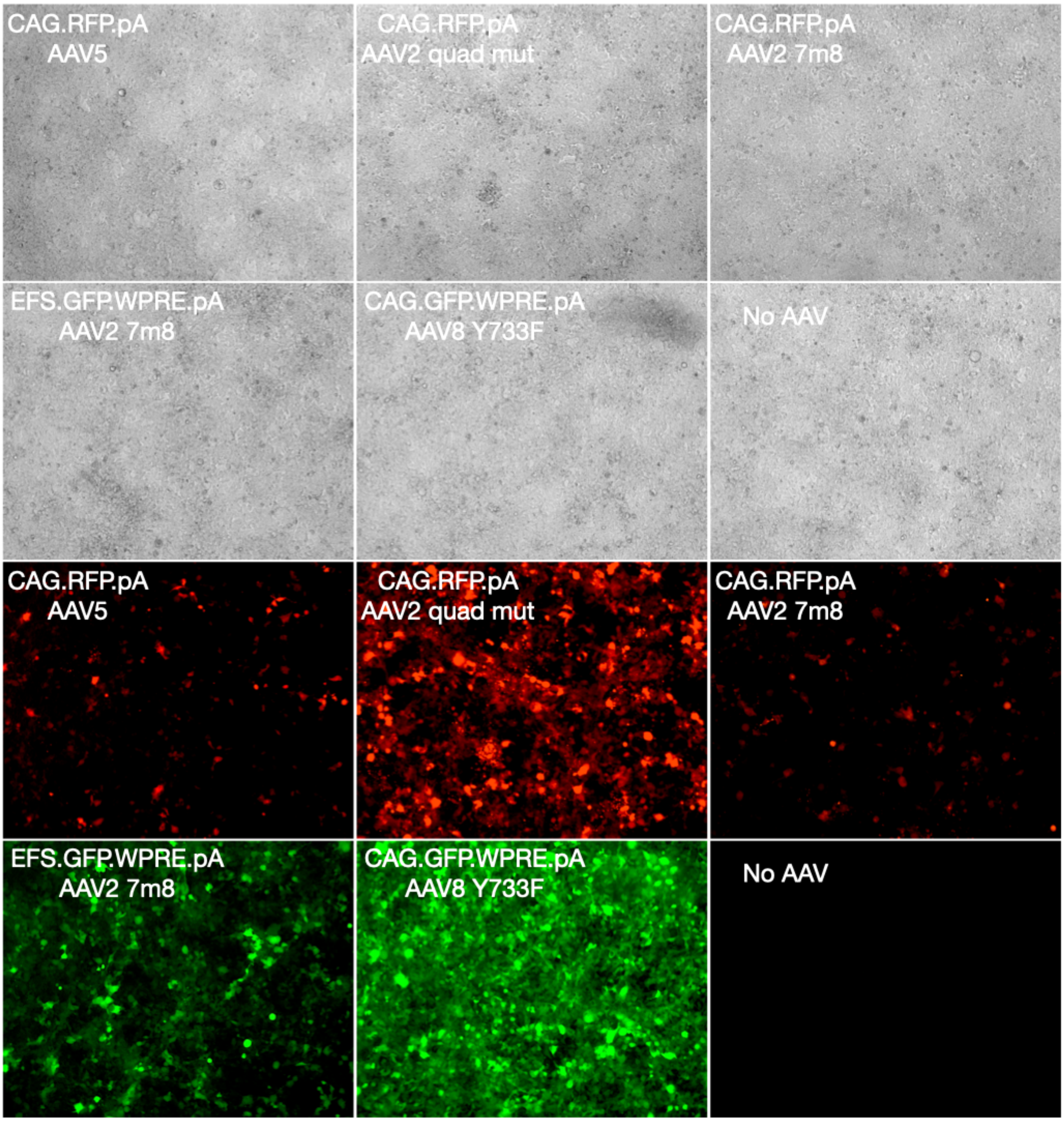

Supplementary Figure 1. *In vitro* testing in HEK293T cells of AAV vectors (MOI 10,000) containing CAG.RFP, CAG.GFP or EFS.GFP reporter transgenes.

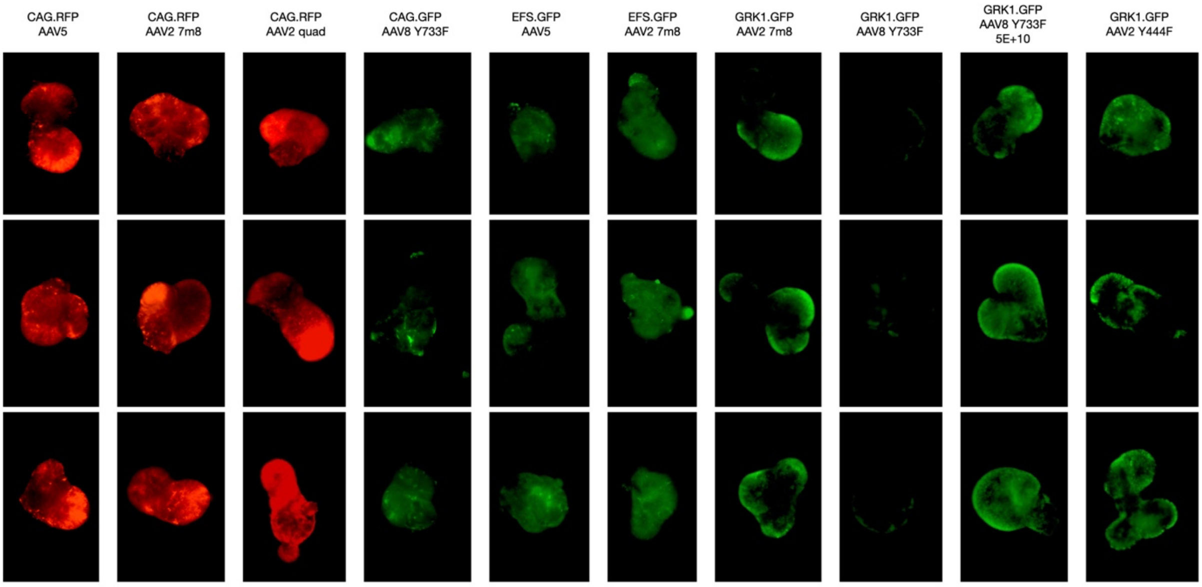

Supplementary Figure 2. Live cell images of three retinal organoids treated with each AAV at 27 days post-transduction.

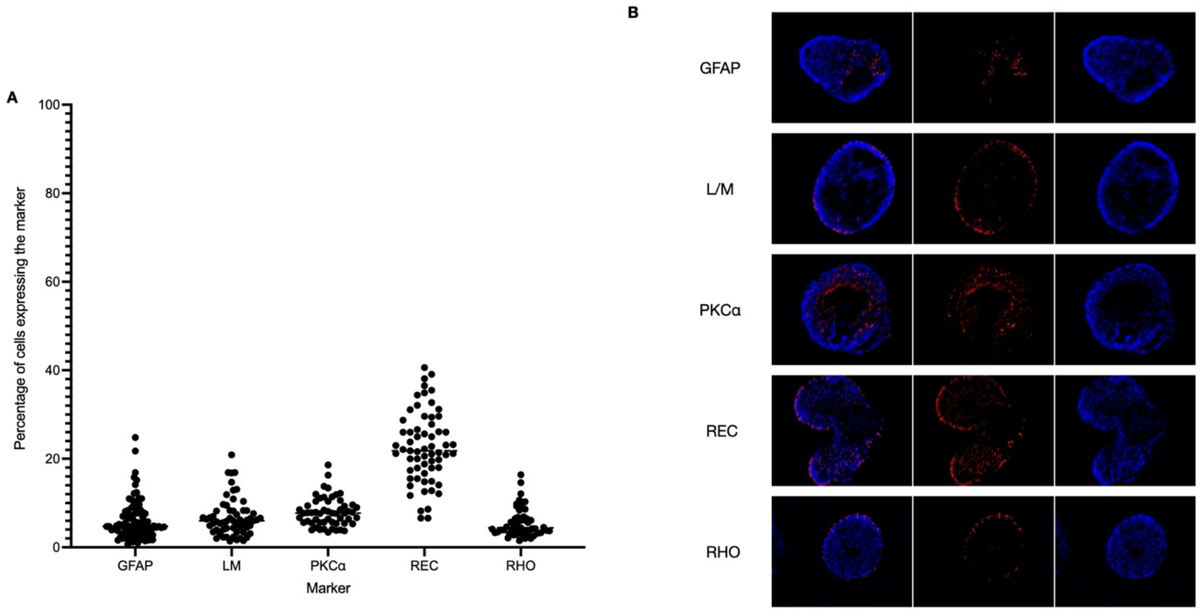

Supplementary Figure 3. Identification of cell types expressing particular markers within the retinal organoids. (**A**) Photoreceptor-like recoverin (REC) positive cells were the most abundant, followed by bipolar-like cells expressing protein kinase C alpha (PKCα), the cone-

like cells expressing L/M opsin (LM), rod-like cells expressing rhodopsin (RHO) and Müller glia-like cells expressing GFAP. (**B**) Representative examples of staining for each marker. A minimum of 55 images were used to obtain the values plotted in (**A**).

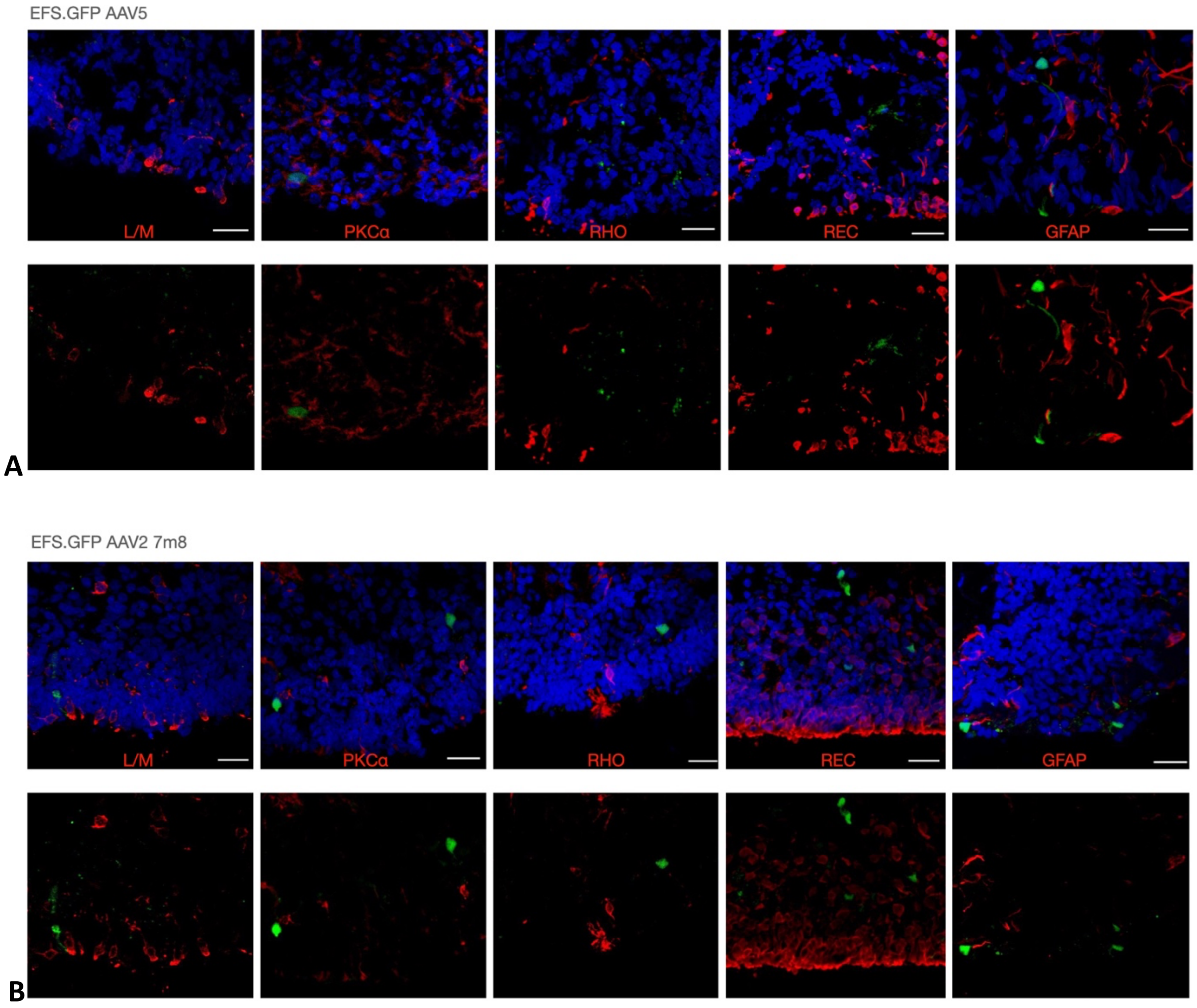

Supplementary Figure 4. Reporter gene expression profiles from EFS transgenes delivered in multiple AAV capsid types. For each sample type, example retinal organoid sections with each marker are shown on the upper panel with only reporter and marker expression in the lower panel, for which overlapping signals are highlighted in white. (**A**) EFS.GFP AAV5 (**B**) EFS.GFP AAV2 7m8. Each retinal organoid was treated with 1E+10 genome copies. Scale bars represent 25µm. GFAP = glial fibrillary acidic protein (Müller glia cell marker); L/M = long/medium

wavelength cone opsin (cone photoreceptor marker); PKCα = protein kinase C alpha (bipolar cell marker); REC = recoverin (pan-photoreceptor marker); RHO = rhodopsin (rod photoreceptor marker).

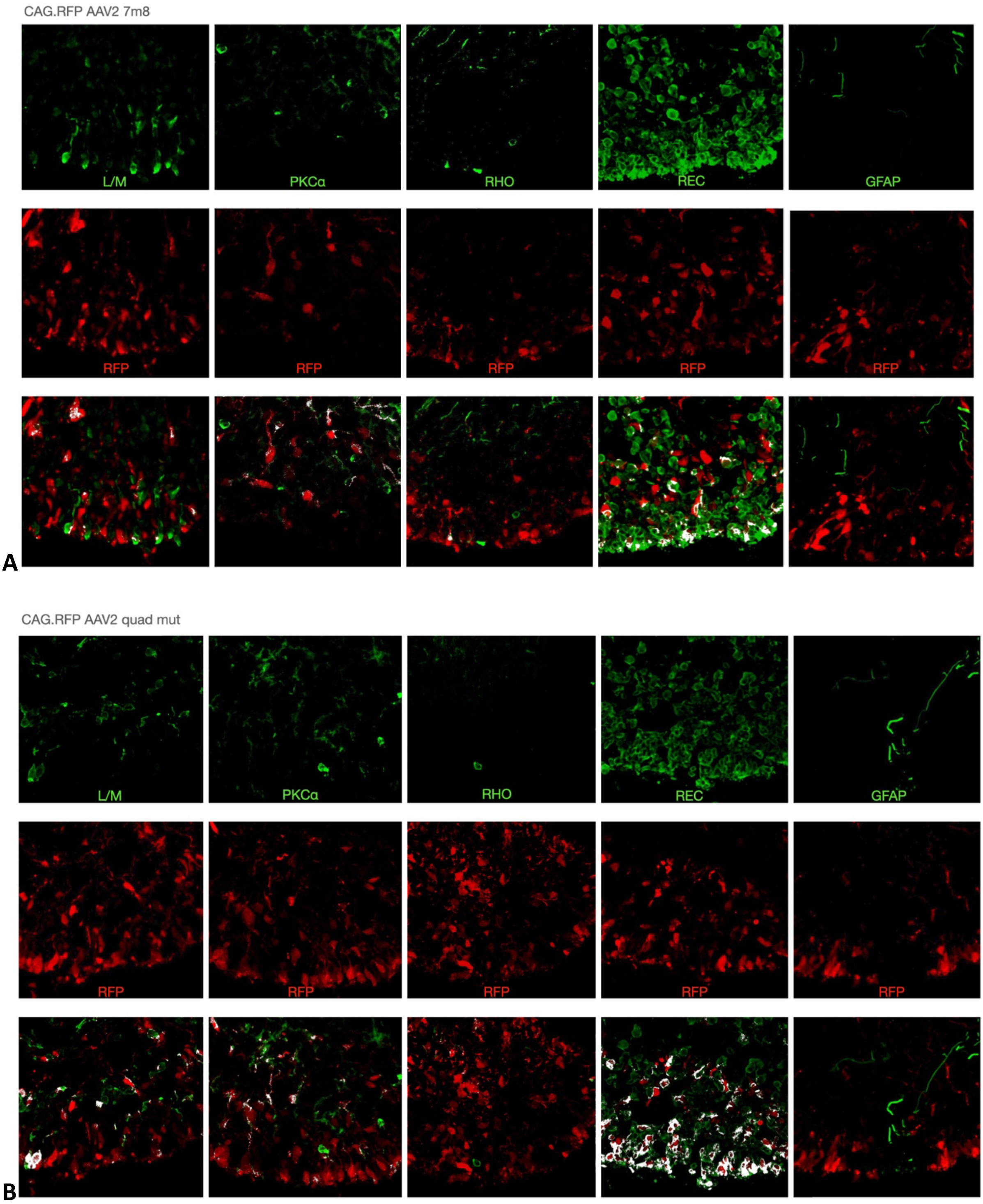

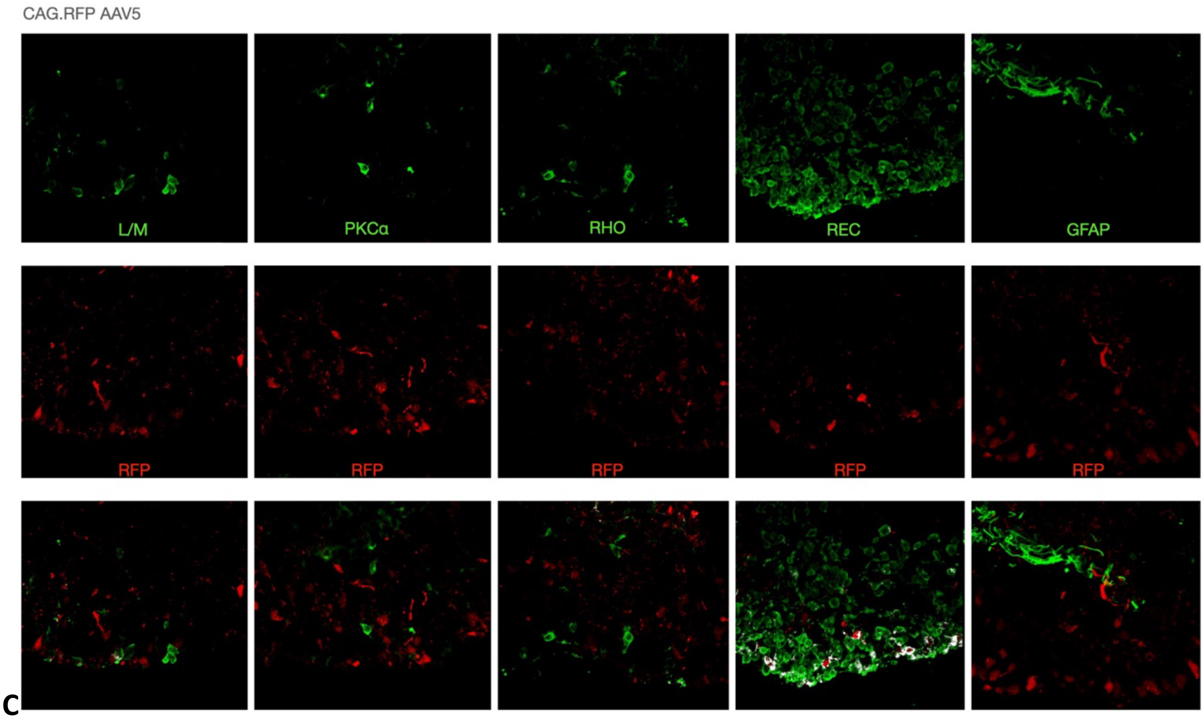

C

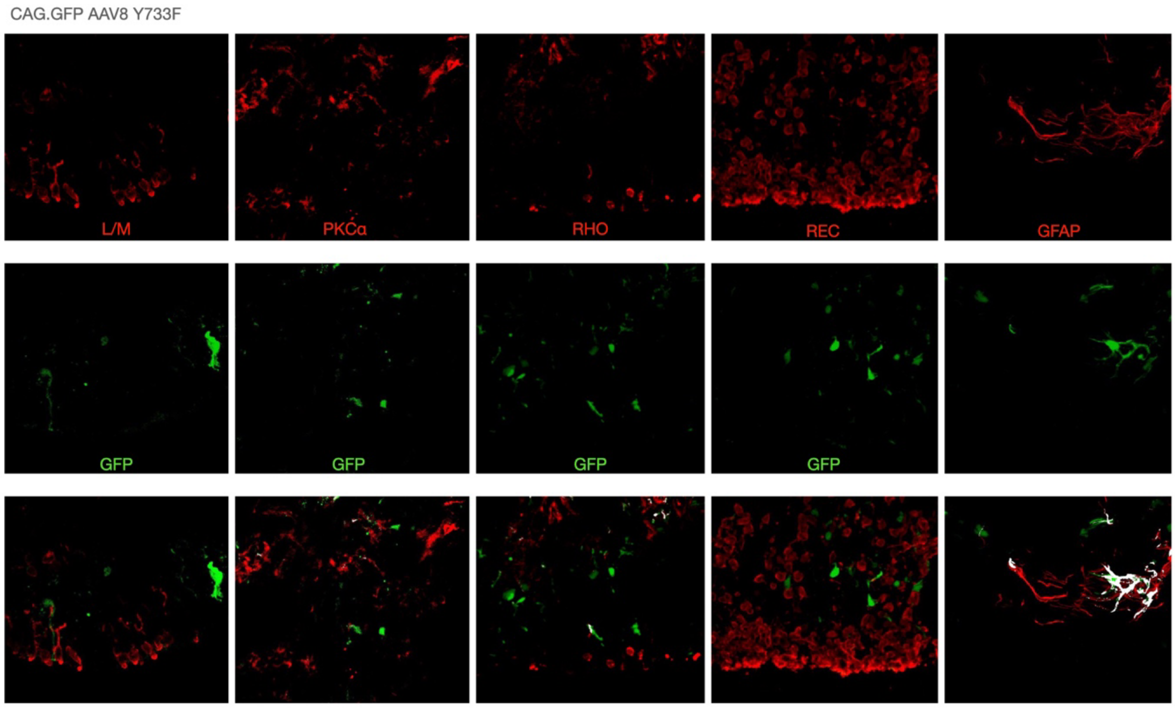

D

Supplementary Figure 5. Representative reporter gene expression profiles from CAG transgenes delivered in multiple AAV capsid types. For each sample type, marker expression is shown on the upper panel with reporter expression in the middle panel and the combined

channels in the lower panel, for which overlapping signals are highlighted in white. (**A**) AAV2 7m8 (**B**) AAV2 quad mutant (**C**) AAV5 and (**D**) AAV8 Y733F. Each retinal organoid was treated with 1E+10 genome copies. GFAP = glial fibrillary acidic protein (Müller glia cell marker); L/M = long/medium wavelength cone opsin (cone photoreceptor marker); PKCα = protein kinase C alpha (bipolar cell marker); REC = recoverin (pan-photoreceptor marker); RHO = rhodopsin (rod photoreceptor marker).

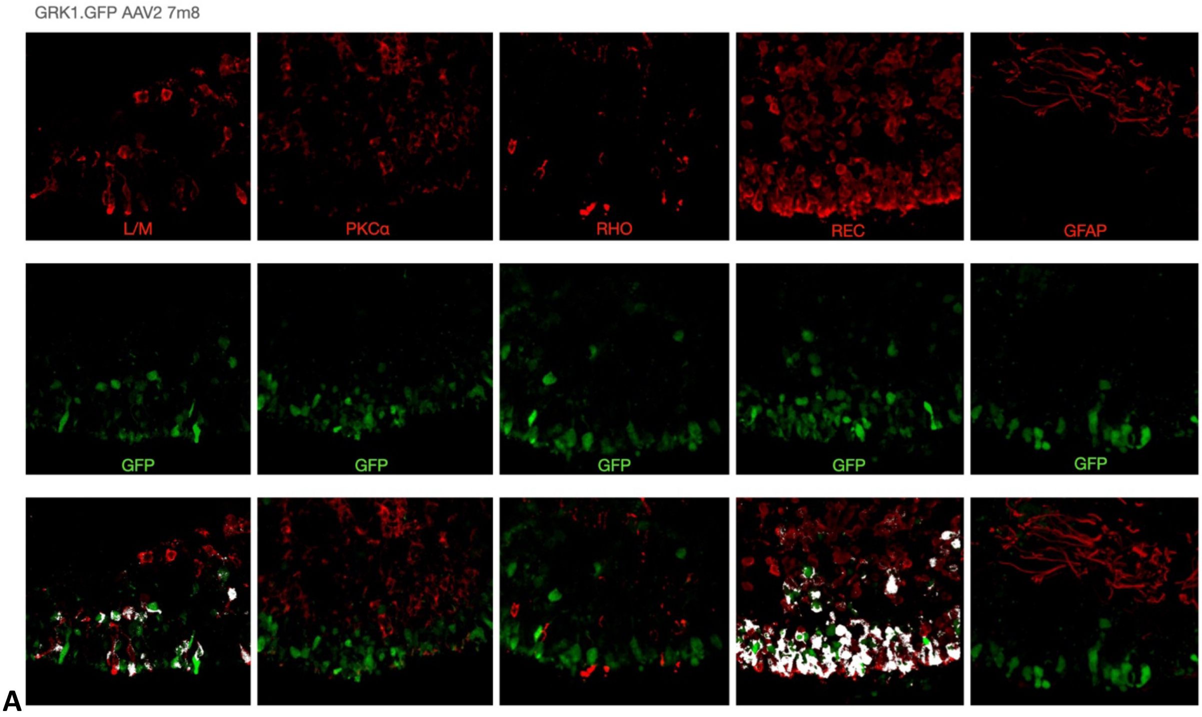

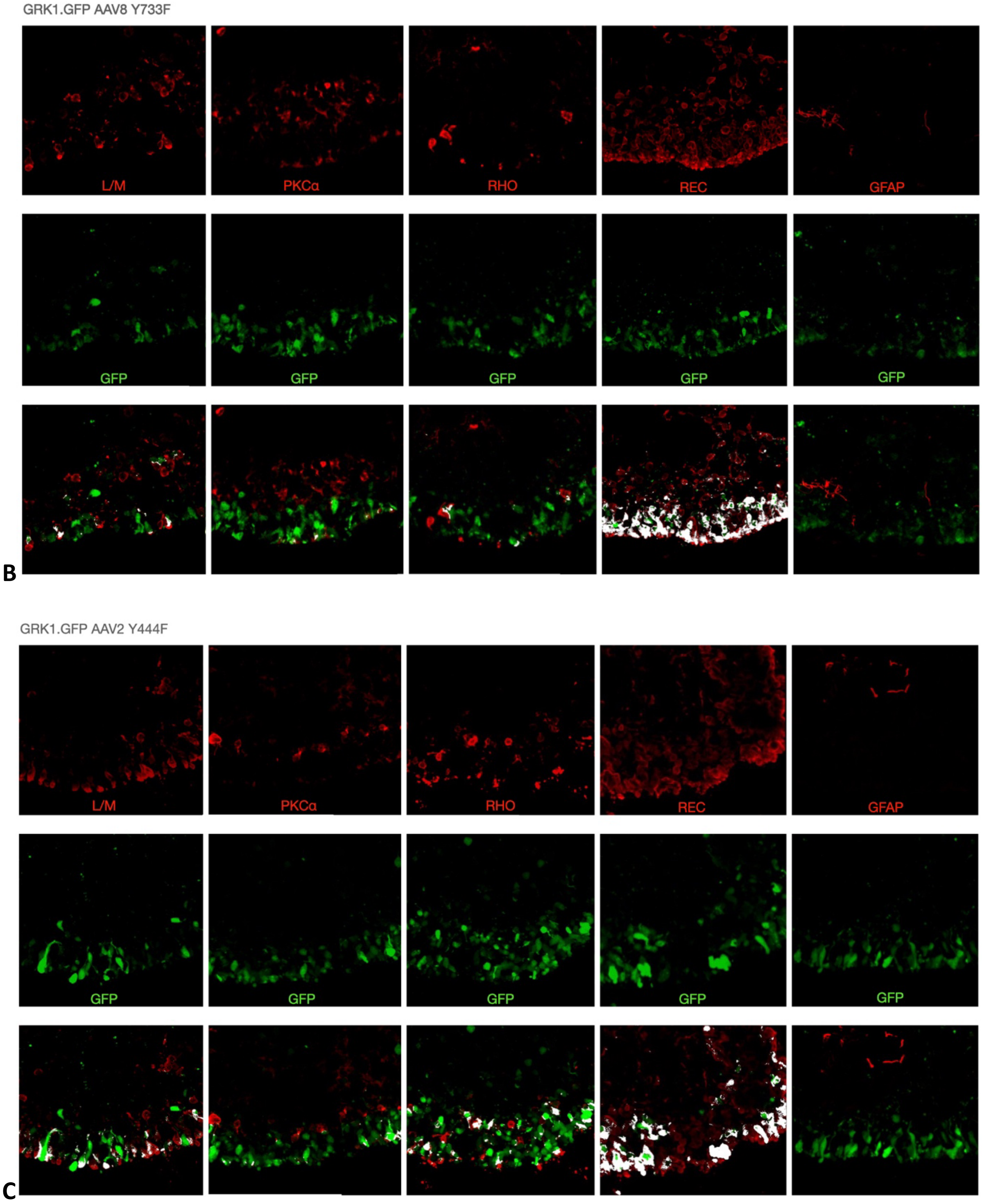

Supplementary Figure 6. Representative reporter gene expression profiles from GRK1 transgenes delivered in multiple AAV capsid types. For each sample type, marker expression is shown on the upper panel with reporter expression in the middle panel and the combined channels in the lower panel, for which overlapping signals are highlighted in white. (**A**) AAV2 7m8 (**B**) AAV8 Y733F and (**C**) AAV2 Y444F. Each retinal organoid was treated with 1E+10

genome copies. GFAP = glial fibrillary acidic protein (Müller glia cell marker); L/M = long/medium wavelength cone opsin (cone photoreceptor marker); PKCα = protein kinase C alpha (bipolar cell marker); REC = recoverin (pan-photoreceptor marker); RHO = rhodopsin (rod photoreceptor marker).

Supplementary Table 1. Co-localisation scores achieved when comparing reporter and marker expression in retinal organoid sections. A minimum of 5 individual Z-stacks from 3 retinal organoids were analysed to provide the average values. Positive transgene/marker correlations are highlighted in bold.

	**GFAP**	**L/M**	**PKCα**	**REC**	**RHO**
**CAG.RFP AAV5**	-0.116	-0.181	-0.187	-0.114	-0.195
**CAG.RFP AAV2 7m8**	-0.044	-0.186	-0.122	-0.132	-0.144
**CAG.RFP AAV2 quad mutant**	-0.049	-0.115	-0.115	**0.043**	-0.112
**CAG.GFP AAV8 Y733F**	-0.099	-0.221	-0.106	-0.125	-0.220
**EFS.GFP AAV5**	-0.137	-0.223	-0.123	-0.225	-0.189
**EFS.GFP AAV2 7m8**	-0.177	-0.162	-0.076	-0.047	-0.255
**GRK1.GFP AAV2 7m8**	-0.030	-0.142	-0.178	**0.105**	-0.122
**GRK1.GFP AAV8 Y733F**	-0.070	-0.031	-0.130	**0.140**	-0.121
**GRK1.GFP AAV2 Y444F**	-0.042	-0.057	-0.110	**0.118**	-0.156
